# Maternal depression symptoms during the first 21 months after giving birth

**DOI:** 10.1177/1403494820977969

**Published:** 2020-12-14

**Authors:** Michael Rosander, Anita Berlin, Karin Forslund Frykedal, Mia Barimani

**Affiliations:** 1 Department of Behavioural Sciences and Learning, Linköping University, Linköping, Sweden; 2 Department of Neurobiology, Care Sciences and Society, Karolinska Institutet, Stockholm, Sweden; 3 Department of Social and Behavioural Studies, University West, Trollhättan, Sweden; 4 Academic Primary Care Centre, Region Stockholm, Stockholm, Sweden; 5 Division of Family Medicine and Primary Care, Department of Neurobiology, Care Sciences and Society, Karolinska Institutet, Stockholm, Sweden

**Keywords:** Maternal depression, postpartum depression, EPDS, screening, transition to motherhood, prevalence

## Abstract

**Aims:**

The first year after childbirth involves a major transition for women, which can accentuate inadequacies and feelings of powerlessness, making them vulnerable to depression. The aim of this study was to investigate the prevalence and frequency of maternal postpartum depressive symptoms at different times after giving birth (0–21 months).

**Methods:**

Data were collected cross-sectionally using a web questionnaire containing the Edinburgh Postnatal Depression Scale (EPDS). A total of 888 mothers with children in the age range 0–21 months responded.

**Results:**

The results showed different levels of depression over the range of months included in the study. The overall prevalence using EPDS ⩾ 12 was 27.8%. There were higher levels at 9–12 months and 17–21 months. The highest levels of symptoms of depression were found at nine, 12, and 17 months after birth, and the lowest levels at two and 16 months.

**Conclusions:**

**Many mothers experience symptoms of depression after giving birth that can continue well beyond the child’s first year. We have identified different levels of depression at different points in time after giving birth, with highs and lows throughout the first 21 months. This highlights a need to screen for depression more than once during the first years, as well as a closer cooperation between midwives and child healthcare nurses in supporting mothers in the transition to motherhood. This is an important aspect of public health, which not only involves mothers with symptoms of depression, but also their ability to care for their child and a possible negative impact on the child’s development.**

## Introduction

The first year after childbirth involves a major transition for all parents [1]. This transition seems to have a larger impact on women as they tend to take a more active role in caring for the child [2]. The new role can accentuate inadequacies and feelings of powerlessness. Environmental factors combined with personality factors, such as low self-esteem and easily being overwhelmed by stress, make a person more vulnerable to depression [3]. Depression is a mood disorder with symptoms affecting how one thinks, feels, and handles daily activities [4]. If the depression occurs within the first year after childbirth, it is labelled a postpartum depression [5] – that is, it is thought of as a depressive disorder with a specific onset. There is a range of names for the kind of depression that occurs in connection with, or as a consequence of, becoming a mother. Postpartum depression is probably the most widely used term, but perinatal or postnatal depression are also used. To include depression that develops later on in early motherhood, maternal depression is sometimes used (see, e.g., Kothari et al. [6] and Woolhouse et al. [7]). In this article, we will use maternal depression when referring to symptoms of depression occurring after birth, as our focus is on the first 21 months. There are different methods used to measure or screen for maternal depression, and the period in focus for when to measure can vary. Measurements are often made within the first 12 weeks postpartum, and in a meta-analysis of 59 studies within that timeframe, O’Hara and Swain [8] found a prevalence of 13%. They excluded assessments from the first two weeks after birth to avoid confounding postpartum blues or “baby blues” (i.e. a mild form of mood disturbance occurring for a majority of new mothers the first few days after birth that is usually resolved by the 10th day [9]). Gavin et al. [10] found a period prevalence of 19.2% for the first three months. In a longer timeframe, up to a year postpartum, approximately 8–15% seem to be afflicted by maternal depression [11,12]. For the majority of women, the depression starts within the first 12 weeks after childbirth [11]. Maternal depression is not only a problem in the prenatal and postpartum period; studies report prevalence of 13.9% at 18 months [6], 11% at 25 months [13], and 14.5% at four years after childbirth [7].

Common symptoms of depression are insomnia, guilt, confusion, emotional lability, dysphoria, and suicidal ideation [11]. What separates depression after childbirth from depression other times in life is that there is a newborn child involved, and a child may risk developing problems of social and emotional functioning [14–16]. There are consequences for a child’s development if the mother suffers from maternal depression – consequences for behaviour, cognitive development, and physical health [17]. O’Hara and McCabe [17] concluded that the severity and duration a child is exposed to the mother’s depression play an important role in predicting future problems. Possible health problems are foremost related to a depressed mother’s reduced ability to care for her child. The negative impact on the child’s development can be balanced by protective factors, such as a competent father or a supporting social environment [18–20]. Unfortunately, some women do not actively seek help for maternal depression [21–25], and healthcare professionals often report low competence in identifying maternal depression [26–29].

One way to detect maternal depression is by screening using the Edinburgh Postnatal Depression Scale (EPDS) [30,31]. It is the most common screening tool used [32]. The EPDS measures depressive symptoms after childbirth, but is not a diagnostic instrument [30]. Many countries screen for maternal depression in well-baby care settings or child healthcare centres (CHCs), with positive evidence showing significantly higher detection rates of maternal depression [33]. However, some studies suggest that EPDS should not stand alone as a screening instrument [29,34]. Tissote et al. [35] argued that maternal depression might be a disorder of its own and not just a depressive disorder in a specific context. They found EPDS to be more sensitive than measures designed to measure major depressive disorder according to The Diagnostic and Statistical Manual of Mental Disorders (DSM-5) [36]. The high sensitivity has also been shown in a review of validation studies of the ESPD [37]. However, the sensitivity might indicate measurement of a wider phenomenon – not only depressive symptoms, but also guilt or anxiety [35].

There is also a question of when to screen for maternal depression. Normally the screening occurs within the first three months after birth [32], but there are indications of a later onset (six to 18 months postpartum) for some women [6]. This means that an early screening would miss those cases. Kothari et al. [6] showed higher levels of depression symptoms at two weeks and 18 months, and lower levels of depression at two and six months. Gjerdingen et al. [38] showed that although there was an initial peak in postpartum depression the first month, a second one was evident at nine months after birth. They used a two-question depression screen (a modified version of the first two questions of the Patient Health Questionnaire (PHQ-9)) as well as the whole PHQ-9. Measuring mothers at the initial well-baby visit at 0–1 month, then two, four and six months at well-baby visits and a questionnaire by mail at nine months. Depression scores were highest at 0–1 month (12.5%) and at nine months (10.2%). A period prevalence for 0–9 months was 22%. Gjerdingen et al. [38] could not explain the second peak at nine months. Different trajectories regarding onset and development have been identified [39,40]. In a study from Brazil, about one in 10 showed an initial high level and then decreasing, whereas a similar proportion showed an increase in symptoms of depression up until 24 months (which was how long the study measured the mothers) [39]. In a study from South Africa, some mothers showed an initial peak at 10 weeks after birth with no symptoms left at six months after birth, but there were also examples of mothers with a peak at 12 months after birth as well as a steady increase up until 18 months after birth [40].

Although there are cohort studies on maternal depression, they normally only measure depression or symptoms of depression at specific points in time – for example, two and eight months for the Avon Longitudinal Study of Parents and Children [41], and six and 18 months for the Norwegian Mother and Child Cohort [42]. As a complement to these types of large cohort studies, the current study, although cross-sectional, contributes to the research field by studying maternal depression symptoms at each month after birth until the child is 21 months old.

## Aim

The aim of this study was to examine the prevalence and frequency of maternal postpartum depressive symptoms at different times after giving birth (0–21 months).

## Methods

### Setting

In Sweden, child healthcare nurses at the CHCs meet all children from birth to six years of age in regular check-ups to see if the child is developing normally. The child healthcare nurse is the key person in the primary child healthcare and has close contact with the parents during the first years of a newborn child. In Sweden, mothers are screened for maternal depression (using the EPDS) 6–8 weeks after childbirth at the CHC by the child healthcare nurse [43].

### Data collection

The study had a cross-sectional design. Data were collected via a web questionnaire. We used a comprehensive questionnaire containing the EPDS [30] together with background questions, but also included other validated instruments measuring, for example, parental stress (the Swedish Parental Stress Questionnaire (SPSQ) [44]) and development of the child (Ages and Stages Questionnaire: Social Emotional [45]). To reach out to a wide range of mothers with a newborn child between 0 and 21 months old, the questionnaire was distributed through the organization “*Föräldravrålet*” (literal translation: “The parental roar”), a non-profit organization in Sweden based on people working for safer childbirth care from a parent perspective. The organization emailed information about the study and a link to the questionnaire to its 7000 members, and also published information and the link on its website (www.foraldravralet.se) and on its Facebook group. Parents who were not members of the organization were also given the opportunity to participate in the study as the information about the study with the link was shared a number of times on Facebook. There was no way of knowing how many of the mothers who belonged to the organization helped distribute the questionnaire, and how many from outside the organization just followed the public links to it. The web questionnaire was open for six weeks from the last week of April 2017.

### Participants

A total of 888 mothers from Sweden who had recently given birth to a child (having a child between 0 and 21 months old) participated in the study. There were also answers from 27 fathers, but they were excluded from the study as the focus was on maternal depression. The mean age was 32.6 years (standard deviation (SD) = 4.22), ranging from 21 to 47 years. Almost all lived in a relationship with a partner (97%). Almost as large a majority of the mothers were born in Sweden (95%). Most of them worked for a living (86%), although most of them were probably on maternal leave at the time of the study. Many had a university degree or were currently taking degrees (77%). About half of the participants lived in larger cities (with more than 250,000 inhabitants), and the other half was evenly spread over varying sizes of smaller cities and towns throughout Sweden. A majority were primiparae (61%), about one third had given birth once before, and the rest (about 10%) had more than two children. Almost half of the mothers had attended some form of parental education group *prior* to giving birth (47%). About three out of four mothers had participated (51%), were currently participating (17%), or were going to attend (5%) a parental education group after giving birth.

The sample is not probabilistic, but for the aim of the study a probabilistic sample is not necessary. We need a relative representativeness of mothers with a child between the age of 0 and 21 months – that is, that those who answered the questionnaire and reported symptoms of depression over the 21 months were not systematically skewed in any other way. This was investigated by use of correlation between the child’s age (0–21 months) and a number of background variables – for example, the mother’s age, education level, total number of children in the family, and size of the city they live in. The correlation between child’s age and all our relevant background variables is close to zero. As information about the data collection was spread using public social media, we also tested for the possibility that the sample consisted of many people other than mothers with a newborn child. If that would be the case, an investigation of the internal structure of the instruments, and the association between EPDS and the other instruments used in the questionnaire, would reveal random answers. All analyses point to a sample containing mothers with a newborn child. The internal structure of the instruments was high, and the correlations between EPDS and other instruments were of an expected size and direction (e.g. the correlation between EPDS and SPSQ was .67).

### Instrument

The instrument used in this study to measure maternal depression was the EPDS [30], which has been validated as a Swedish translation and in a Swedish context [25,46–48]. The EPDS comprises 10 items (e.g. “I have been able to laugh and see the funny side of things”, with a response scale from 0, “As much as I always could”, to 3, “Not at all”) and is scored using the sum of the items (each scored 0–3) on a scale from 0 to 30 – the higher the score, the greater the risk of depression. The internal consistency of EPDS in this study was good (Cronbach’s alpha = .87). A cut-off score of ⩾ 10 has been recommended by some studies [22,33–34,49] for both minor and major depression, while ⩾ 12 has often been used for screening for major depression [34]. Other cut-off scores have also been used – for example, ⩾ 13 [7,11,35]. The Swedish translation of EPDS has been validated using ⩾ 12 as the cut-off [25], which is also the recommended cut-off used in the clinical practice in Sweden [43]. Based on this, the cut-off score used in this study was EPDS ⩾ 12.

### Other variables

The age of the mother, age of the newly born child, and number of children in the family were also collected. When focusing on frequency in relation to the cut-off score on EPDS, the variables were grouped for clarity. The rationale behind the division was mainly to get measurements from the 22 months of the study into a more apprehensible format. We strived to have as equal groups as possible in terms of equal division of months as well as equal total size of each age category. This resulted in five categories for the age of the newly born child (0–4, 5–8, 9–12, 13–16, 17–21 months).

The mothers’ experiences from participating in parental education groups were also used in the study. The variable capturing experiences prior to giving birth was dichotomous – having experience or not. For experiences from parental education groups after giving birth, there were four possible categories: not having any experience (and no plan to participate), future participation (as the group had not started yet), ongoing experience, and past experience from a parental education group. The variable was dichotomized – having experience (ongoing or past) or not.

### Data analyses

Data were analysed using the statistical software Stata 15.1 for Mac. χ^2^ was used to investigate differences in frequency, correlation, as well as *t*-tests to compare differences for education level, city size, and experience from parental education groups. To be able to control for the background variables, a logistic regression was used to show the odds ratio (OR) for the different child age categories on the EPDS score (cut-off).

### Ethical considerations

All participants were informed of the overall aim of the study, and it was clarified that it was voluntary and that anyone could drop out from the study at any time. Answering and submitting the web questionnaire was regarded as consent to being a part of the study. As the participants were completely anonymous to us, it was not possible to contact or follow-up on mothers with high scores. The participants were informed about their anonymity. The study was approved by the regional Research and Ethics Committee at Linköping University, Sweden (#2017/202-31).

## Results

### Prevalence of maternal depression symptoms

**Figure 1.**
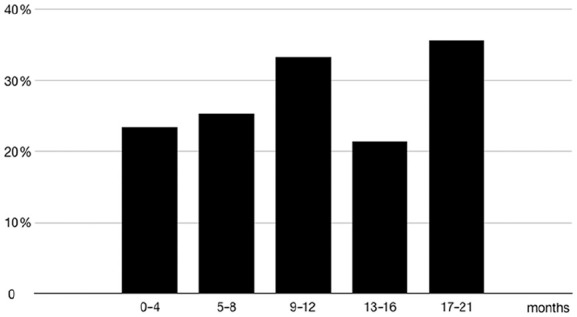
Percentage of mothers scoring above the cut-off (EPDS ⩾12) over five age categories.

The overall period prevalence (0–21 months) of maternal depression symptoms in this sample was 27.8% for EPDS ⩾ 12. In Figure 1, the percentages of mothers above the cut-off score for different ages of the newborn child (in five age categories) are presented. There was a higher level of depression symptoms at 9–12 months and at 17–21 months. A logistic regression showed an almost doubled risk of having an EPDS score above the cut-off for these two age categories (OR 1.88 and 2.02), controlling for the age of the mother, number of children, family situation, education, employment, city population, place of birth, and having received parent education before and after giving birth (see Table I). Based on the variables used as control, only those on sick-leave, a category in the variable Employment, showed a significant heightened risk of having an EPDS score above the cut-off (OR 6.2, *p* = .044, confidence interval (CI) 95%: 1.05–36.32). That result is not surprising, but it involves a very small group of mothers (0.8% of 888). The mothers not currently participating or having participated previously in parental groups showed a close to significant heightened risk (OR 1.4, *p* = .053, CI 95%: 0.99–1.98).

**Table I.** Logistic regression predicting EPDS ⩾ 12.

Age of child	*N*	EPDS ⩾ 12				
		*n*	%	OR	CI 95%	*p*-value
0–4 months	218	51	23.4%	1	Base	
5–8 months	174	44	25.3%	1.24	0.76–2.03	ns
9–12 months	207	69	33.3%	1.87	1.18–2.94	.007
13–16 months	140	30	21.4%	1.08	0.63–1.84	ns
17–21 months	149	53	35.6%	2.02	1.24–3.30	.005

Covariates: age (mother), number of children, family situation, education, employment, city population, place of birth, parent education before and after giving birth.

ns: not statistically significant.

**Figure 2.**
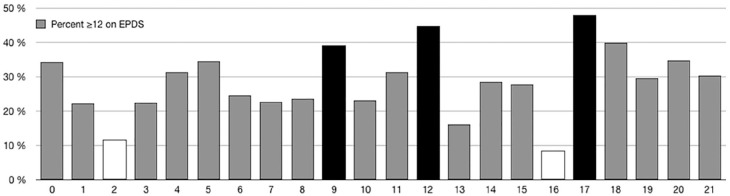
Frequency of maternal depression at different ages of the newborn child (0–21 months). Black bars represent significantly higher than expected frequencies and white bars lower than expected frequencies.

The age of the mother showed no difference in prevalence(χ^2^(5) = 6.95, *p* = .224), although the youngest mothers (21–28 years) showed a somewhat higher percentage than the other age groups (35% compared to between 22% and 29% for the others). The correlation between EPDS score and the mothers’ age was close to significant: *r*(877) = −.06, CI 95% (–.13; .00). The correlation is small but negative, indicating a small decrease with age. The age of the newly born child did show differences in frequency of maternal depression symptoms (χ^2^(21) = 36.01, *p* = .022). In Figure 2, the frequency at the different child ages is presented. The analysis showed a higher than expected frequency for mothers with children aged nine, 12, and 17 months, and a lower than expected frequency for mothers with children aged two and 16 months. In the Table II frequencies, percentages, χ^2^ contribution for each aaaaa, means, SD, and minimum and maximum for ages 0–21 months are presented.

**Table II.** Frequency and percentage of mothers having a newborn child aged 0–21 months with EPDS ⩾ 12, χ^2^ contribution for each aaaaaaaa, mean EPDS, SD, and minimum and maximum scores.

Age (youngest child, months)	*n*	Freq EPDS ⩾ 12	Percent EPDS ⩾ 12	χ^2^ contribution	Mean EPDS	SD	Min	Max
0	32	11	34.4%	0.5	9.66	4.31	3	21
1	36	8	22.2%	0.4	7.53	4.74	1	18
2	53	6	11.3%	5.2	7.42	4.03	0	20
3	49	11	22.4%	0.5	7.80	5.24	0	26
4	48	15	31.2%	0.2	9.44	5.37	0	21
5	26	9	34.6%	0.4	8.85	5.47	1	24
6	49	12	24.5%	0.2	8.24	4.80	0	20
7	44	10	22.7%	0.4	8.50	4.96	1	24
8	55	13	23.6%	0.3	7.74	4.74	0	24
9	49	19	38.8%	2.1	9.24	6.00	0	24
10	65	15	23.1%	0.5	8.72	5.08	2	27
11	48	15	31.2%	0.2	9.85	5.91	0	24
12	45	20	44.4%	4.5	10.93	5.51	1	27
13	37	6	16.2%	1.8	7.84	5.36	1	24
14	42	12	28.6%	0.0	8.88	5.00	1	24
15	36	10	27.8%	0.0	9.08	4.56	2	19
16	25	2	8.0%	3.5	6.84	4.67	0	21
17	21	10	47.6%	3.0	11.00	4.64	2	21
18	25	10	40.0%	1.3	10.76	5.04	1	21
19	37	11	29.7%	0.0	8.94	4.87	0	19
20	43	15	34.9%	0.8	8.88	5.98	0	25
21	23	7	30.4%	0.1	8.96	4.46	2	16

The frequency at about two months, which is when EPDS is normally administrated to mothers by child health nurses, was 11.3% (*n* = 53) and the second lowest score for the whole period 0–21 months after birth. There was no difference at all in prevalence based on participation in parental groups *before* giving birth: (χ^2^(1) = 0.00, *p* = .988). However, experience from parental groups *after* giving birth showed a difference in prevalence: 25.6% for those having participated and 32.6% for those without experience (χ^2^(1) = 4.67, *p* = .031). There were no differences comparing prevalence for those with a university degree (27.7%) with those with less education (27.8%), or for those living in big cities (28.2%) with those from smaller towns (27.6%). The same result was found comparing mean EPDS scores between education levels (*t*(885) = 0.27, *p* = .785) and size of the city (*t*(885) = −0.38, *p* = .704). There were no differences comparing prevalence for primiparae mothers (26.5%) with those with more than one child (29.7%) (χ^2^(1) = 1.08, *p* = .297), or comparing mean EPDS scores (*t*(884) = −0.90, *p* = .367).

## Discussion

The aim of this study was to investigate the prevalence and frequency of maternal depressive symptoms at different times after giving birth. The results showed a rather high level of maternal depression symptoms – overall, nearly 28% (EPDS, cut-off ⩾ 12) for the whole period of 0 to 21 months after birth. This can be compared to the 22% 0–9-month period prevalence of maternal depression symptoms found by Gjerdingen et al. [38] and Gavin et al.’s [10] 0–3-month period prevalence of 19.2%. The results of the current study showed higher levels at nine, 12 and 17 months after childbirth, and a doubled risk of depression symptoms at 9–12 months and at 17–21 compared to the other times. This is an interesting find, which adds to the growing evidence of maternal depression occurring not only in the first few months after birth [6,38–40]. Both Kothari et al. [6] and Gjerdingen et al. [38] found high levels of depression symptoms in the first month. This could possibly have been diluted by the “baby blues” normally ending after 10 days [9]. The blues could also have contributed to the scores of the first months of the current study. Kothari et al. [6] found lower levels of depression symptoms at two and six months after birth, but then another high at 18 months. By choice of measurement time, while showing the lows at two and six months, it is possible they missed the opportunity to identify the high levels found in the current study at around nine months and the low level of depression symptoms at about 16 months after childbirth. In a similar vein, Gjerdingen et al. [38] found an increase in symptoms of depression at nine months but did not include any measurements after that point.

The reasons for the high and low levels of depression symptoms are not altogether clear. Kothari et al. [6] identified a group of women with a later onset of depression symptoms that could possibly also be found in the current sample and contributing to the higher level at 17 months postpartum. Another reason may come from the fact that in Sweden a parent has the right to stay at home with one’s newborn child until it is 18 months old [50]. As depression can stem from a stressful life event [5], it is reasonable to see a higher level of depression symptoms as a result of a new life phase for many mothers, involving going back to work and finding new ways to relate to one’s child. However, it does not explain the low level at 16 months (the lowest score of all in the study). The low level found at two months postpartum could be related to both a reduction or absence of the postpartum blues [9], and having adjusted to the daily routines of a new mother. By six weeks after childbirth, most women also have completed the physiological transition and established nutrition [51], which could contribute to the understanding of the lower scores at two months.

At about 7–10 months, with the development of person (mother) permanence, the child may be wary of unfamiliar humans, becoming sad and worried if the mother leaves the room [52]. For the mother, this can result in feelings of being tied down and a greater difficulty in being relieved by another care-person. This could be one reason for the higher values at 9–12 months. By 12 months, many mothers are getting back to work, which might also contribute to the higher levels of depression symptoms during this period compared to other times (national statistics show an increase one year after childbirth in the number of parental leave days the father uses [53]).

The current study, although not longitudinal, has shown different levels of depression symptoms during the first 21 months after childbirth. This suggests a need to screen for depression more than once. The time of screening currently used, at 6–8 weeks after childbirth, also corresponds to the point in time with the lowest scores, both in the current study, but also in the studies by Kothari et al. [6] and Gjerdingen et al. [38]. Only focusing on maternal depression at this point in time probably makes screening a blunt tool only showing a fraction of the mothers in need of support. This is also supported by Woolhouse et al. [7], who found high levels of depression symptoms both at 18 months and at four years postpartum. There was a doubled OR for women with only one child at this point, so the increase could not be explained by reaching another “peak time of vulnerability to depression” (p.316) as it has been thought of previously. As a counterpoint, Knights et al. [49] argued for not repeating an early negative screen (within 96 hours of delivery) for all mothers, as the likelihood of changes later on is small. This accentuates the need to also focus on known risk factors such as prior depression, poor social support, history of substance abuse, and being an adolescent mother [10,49].

A small, close to significant, negative correlation between EPDS score and age of the mother indicates a small decrease in maternal depression symptoms with age. However, this can be understood as somewhat higher levels of depression symptoms for the youngest mothers compared to the other mothers. This could possibly have been clearer if even younger mothers would have been part of the study, as adolescent mothers are more exposed to stressors that could make symptoms of depression more pronounced, such as a greater risk of isolation from peers and low self-esteem [54].

An interesting find was that having had parental education, being part of a parental group, before giving birth seems to have had no effect on the level of maternal depression. However, having experience from a parental group after giving birth had a small but significant positive effect on the symptoms of depression. One function of a parental group after giving birth is to provide a new social network with other parents, which could help reduce social isolation that otherwise can be a risk factor for maternal depression [10].

Both midwifes and child healthcare nurses are health professionals who are close to the women after childbirth. One of their tasks is to identify women with symptoms of depression [5,24], and their regular contact is highly important to build a trusting relationship in order to find those women who do not actively seek help for their depression. Routinely conducted psychosocial assessments such as the EPDS are discussed in the midwifery practice [29] as well as in the child healthcare practice [13,33]. However, as argued by Matthey [55], there is a risk of overestimating the true rate of depression using screening tools such as the EPDS, and thereby a risk of neglecting a focus on mothers who really need support for issues of maternal distress. As, for example, prenatal depression and social support are important predictors of maternal depression [56], the importance of a closer interprofessional teamwork between midwives and child healthcare nurses to identify social health concerns and emotional needs for women and their families is highlighted [57,58].

A limitation of the study, which could also be one of its strengths, is that the data collection was not done using the CHC as a channel for getting participants. Instead, we used a large non-profit organization that we knew had many new mothers as members, and a snowball method to help spread the information about the study, hoping to reach even more new mothers that way. A risk with this strategy is that other people answer the questionnaire by just stumbling over the web link to it. The probability of getting sensible data with many not from the intended sample would be low, and none of our tests reported in the methods section indicated this. There are possible advantages of not going through the CHC, as doing so might give ideas to participating mothers that the results of symptoms connected to a stigma such as maternal depression might get into their medical record. Other studies on maternal depression guarantee confidentiality and sometimes anonymity also via the CHC, but just knowing they are singled out as a possible participant by their CHC might influence how a mother approaches answering questions about their mental health all the same. In our study, participants were completely anonymous to us.

Another limitation of the current study was that it was cross-sectional and not longitudinal. This means we do not have information about the onset of depression, only that the levels of depression symptoms were at certain levels at certain times after birth. We cannot see changes in depression symptoms over time, only that at certain months after birth the mothers representing those months reported more symptoms of depression than mothers representing other months after birth. However, the results correspond to other studies using a longitudinal design. For example, Kothari et al. [6] showed trajectories indicating both an early and a late onset of depression symptoms, and Gjerdingen et al. [38] showed a second peak at nine months after an initial peak at 0–1 months. This suggests that our results are reasonable despite having a cross-sectional design. One strength of the study is that we had many participating mothers and data from each month from 0 up to 21 months after birth, not only 2–3 times during the postpartum period. The selection was probably not representative for Swedish mothers, as the organization providing contact to many of the participants probably attracts more educated mothers and not mothers from all walks of life. This was also apparent in that the youngest mothers in the study were 21 years of age. A self-selected sample may be skewed in terms of depression symptoms compared to the general population, and this has an impact on the external validity of the findings of the study in terms of the magnitude of the levels of depression symptoms. However, the impact on the internal validity should be smaller – internal validity in terms of the levels of depression symptoms comparing mothers within the sample who have children at different ages. There is no reason to assume anything other than that the distribution of months after birth was random. So, although the levels of depression symptoms may not be generalizable to the population of mothers, the differences between different months after birth could help understand symptoms of depression at different times for new mothers. Also, there were no significant differences in level of depression when comparing, for example, educational level.

The current study used a web questionnaire for data collection and the mothers were not known to us. This brings up the question of whether filling out a form about depression knowing one’s nurse will know the result, and that it will appear in medical records, leads to a more cautious approach, which could help explain the somewhat higher levels of maternal depression symptoms found in the current study compared to many other studies. We used an EPDS score of 12 or higher as the cut-off score in this study. One reason for this, as we included scores for mothers from within the first few weeks of delivery, was that a lower cut-off score would probably be more exposed to the risk of dilution because of the baby blues in the answers of those mothers.

## Conclusion

The transition to becoming a mother can be difficult due to changes in the parental role and family relationships, as well as psychosocial adaptations, changes in self-perception, and body image. Many mothers experience symptoms of depression after giving birth that can continue well beyond the child’s first year. We have identified different levels of depression at different points in time after giving birth, with high levels and lower levels throughout the first 21 months after birth. The results of the study indicate a need to screen for depression more than once during the first years. However, more studies using a high frequency of measurement with a longitudinal design are needed to verify and more precisely show the optimal point in time for subsequent testing. To be better able to support mothers in the transition to motherhood, a closer cooperation between midwives and child healthcare nurses could also possibly help identify women at risk of becoming depressed. A better use of parental groups after birth could probably also help in reducing the number of mothers experiencing symptoms of depression by providing a social context and possibilities for networking with others in the same position, reducing the risk of becoming overwhelmed by all the new things that come with parenthood.
